# Plasmolysis-deplasmolysis causes changes in endoplasmic reticulum form, movement, flow, and cytoskeletal association

**DOI:** 10.1093/jxb/erx243

**Published:** 2017-08-23

**Authors:** Xiaohang Cheng, Ingeborg Lang, Opeyemi Samson Adeniji, Lawrence Griffing

**Affiliations:** 1 Biology Department, Texas A&M University, TAMU, College Station, TX, USA; 2 Cell Imaging and Ultrastructure Research, University of Vienna, Althanstrasse, Vienna, Austria

**Keywords:** Cytoplasmic streaming, endoplasmic reticulum, Hechtian reticulum, Hechtian strand, membrane contact sites, persistency mapping, plasmolysis

## Abstract

Plasmolysis of hypocotyl cells of transgenic *Arabidopsis thaliana* and *Nicotiana benthamiana* diminishes the dynamics of the remodeling of the endoplasmic reticulum (ER) in the central protoplast, namely that withdrawn from the cell wall, and more persistent cisternae are formed, yet little change in the actin network in the protoplast occurs. Also, protein flow within the ER network in the protoplast, as detected with fluorescence recovery after photobleaching (FRAP), is not affected by plasmolysis. After plasmolysis, another network of strictly tubular ER remains attached to the plasma membrane-wall interface and is contained within the Hechtian strands and reticulum. FRAP studies indicate that protein flow within these ER tubules diminishes. Actin is largely absent from the Hechtian reticulum and the ER becomes primarily associated with altered, branched microtubules. The smaller volume of the central protoplast is accompanied by decreased movement rates of tubules, cisternae, and spheroid organelles, but this reduced movement is not readily reversed by the increase in volume that accompanies deplasmolysis.

## Teaser

Upon plasmolysis, protoplast ER becomes more persistently cisternal without changing its actin association or internal flow, while wall-associated ER is tubular, associated with altered microtubules, and has reduced flow.

## Introduction

Plasmolysis is generally a reversible decrease in the volume of a walled plant cell protoplast caused by water flow down a gradient along the chemical potential of water when the cell is exposed to hyperosmotic external solute concentrations. In highly vacuolated cells, such as the elongated hypocotyl cells examined here, this decrease in cellular volume comes primarily from water transport out of the vacuole and is achieved when the water potential outside the cell is lower than that necessary to abolish the hydrostatic pressure of turgor, namely the point of incipient plasmolysis, resulting in volume change as water flows out of the protoplast and it shrinks away from the surrounding wall (Nobel, 2009). Shrinkage away from the wall is, however, limited in many cell types by a physical linkage between the plasma membrane (PM) and the wall, a linkage that is strong enough to remain intact even as the bulk of the protoplast shrinks. If the wall-PM anchor sites are small, then plasma membrane tubules as small as 50–100 nm in diameter (Oparka *et al.*, 1994) connect the withdrawn protoplast to the wall and are called Hechtian strands (Hecht, 1912). If the anchored area of the plasma membrane is large, then the unanchored plasma membrane of the shrinking protoplast retracts around the anchored area, producing a thin plasma membrane ‘sandwich’, around 100–200 nm in thickness, which under light microscopy appears tubular or cisternal and is called the Hechtian reticulum.

Hechtian strands and the Hechtian reticulum have both been labeled with DiOC_6_ (Oparka *et al.*, 1994; Lang-Pauluzzi and Gunning, 2000), a carbocyanine fluorescent dye that accumulates in response to membrane potential (Terasaki, 2000). Although its selectivity for different organelles varies, it is often used to stain the mitochondria and endoplasmic reticulum (ER). Furthermore, some, but not all, Hechtian strands form at plasmodesmata, where the plasma membrane is not only attached to the wall but traverses it, and is associated with the ER desmotubule within the plasmodesmata. The ER attached to the desmotubule is presumably within these Hechtian strands. However, because DiOC_6_ can be a non-specific label (Terasaki *et al.*, 2001) and not all Hechtian strands form at plasmodesmata, the question of the ubiquity of ER in the Hechtian strands and Hechtian reticulum has been unresolved. Here, we show that ER is in both the Hechtian strands and Hechtian reticulum using plants expressing ER-localized fluorescent proteins with carboxy-terminal ER retention signal (HDEL) fusions.

Analysis of the ER morphology in the protoplast after plasmolysis was examined using persistency mapping (Sparkes *et al.*, 2009; Griffing *et al.*, 2014), whereby those tubular or cisternal features that persist for longer than 1.5–5 s are analyzed. Increases in persistent ER cisternae are characteristic of treatments that interfere with myosin XI-K, 1, and C, (Myo11E, Myo11F and Myo11C1; Park and Nebenfuhr, 2013; Griffing *et al.* 2014). In young and therefore small hypocotyl and root cells the ER is also more cisternal (Ridge *et al.*, 1999). In addition, young and small cells have slower rates of streaming than older, large cells (Stefano *et al.*, 2014). Can changing the size of a cell’s protoplast change the form of its ER? In this study, we show that in addition to decreasing the rate of streaming within the cell, persistent ER cisternae also increase upon plasmolysis. However upon deplasmolysis, rates of streaming remain low and a high level of persistent cisternae occurs, uncoupling these events from cell size.

The flow within the ER, as distinct from remodeling or translational movement of tubules or cisternae, has been shown to be relatively insensitive to drug treatments that depolymerize microtubules and actin microfilaments and to treatments that interfere with myosin XI-K activity (Sparkes *et al.*, 2009). Consequently, although there may be an underlying organizational role for the cytoskeleton, the directional flows that occur within the plant ER (Runions *et al.*, 2006) may be more influenced by other factors. We also find that a plasmolysis-deplasmolysis cycle does not change flows within the ER in the protoplast but does change the flow in the Hechtian strands and reticula.

Likewise, the association of cortical ER with the cytoskeleton changes in the Hechtian reticulum. Most cortical ER dynamics are regulated by the actin-myosin system and not by microtubules, since latrunculin B, but not oryzalin, increases the persistency of tubules and cisternae (Sparkes *et al.*, 2009). There are some instances where cortical ER can track on microtubules in the presence of latrunculin B (Hamada *et al.*, 2014; Griffing *et al.*, 2016) and these events are much less dynamic than the actin-myosin mediated movement (Sparkes *et al.*, 2009; Ueda *et al.*, 2010). However, as described below, we find that the ER remaining in the Hechtian reticulum colocalizes with altered, branched microtubules found there and the ER tubules appear to track exclusively along these altered microtubules and not the filamentous actin network.

## Materials and methods

### Transgenic plant growth and plasmolysis

*Nicotiana benthamiana* and *Arabidopsis thaliana* seedlings were grown in half-strength modified basal salts of Murishige and Skoog media (Caisson Laboratories Inc., North Logan, UT USA) with 1% (w/v) agar at room temperature in 17.5-hour-long days for 7 to 14 d. For tobacco experiments, *N. benthamiana* line 16c (Haseloff *et al.*, 1997; Ruiz *et al.*, 1998) was used. It constitutively expresses GFP-HDEL in the ER lumen. For Arabidopsis experiments, several different transgenic lines were used. Arabidopsis constitutively expressing the GFP-tagged actin-binding domain of fimbrin1 (GFP-FABD) was used (Voigt *et al.*, 2005); seeds were generously gifted by Prof. Jozef Samaj. In addition, transgenic Arabidopsis plants constitutively expressing a double YFP-fusion to the carboxy and amino terminal regions of the actin binding domain of fimbrin (35S: YFP-ABD2-YFP, courtesy of Elison Blancaflor, Noble Foundation, Ardmore, OK, USA) and a GFP-TUA6 labeling alpha-tubulin (Ueda *et al.*, 1999) were used. The latter two Arabidopsis lines were transformed by floral dip with Agrobacterium with a binary vector containing a signal-transit-sequence-mCherry-HDEL fusion re-engineered from a yeast plasmid courtesy of Eric Snapp, Janelia Research Campus, Ashburn, VA, USA. Individuals expressing strong fluorescence in the T2 generation were identified prior to experimentation.

Plasmolysis experiments were carried out on whole seedlings mounted in 10 mM 2-(N-morpholino) ethanesulfonic acid (MES) buffer (Sigma-Aldrich, St. Louis MO USA, pH 5.8) without (control) and with plasmolyticum (0.75 M sorbitol). Following analysis without plasmolyticum, the MES buffer was completely removed by absorption with Kimwipes (Kimtech Science, USA) at the edge of the coverslip, followed by the addition of plasmolyticum to completely immerse the seedling. Seedlings lose turgor during the first 15 min of hypertonic solution treatment, causing a loss of focal plane, which was then adjusted. To minimize frame shifting and loss of focus, some of the experiments were performed with a coverslipped seedling in 35 mm uncoated No. 1.5 glass-bottom dishes (made in-house).

### Fluorescent dye labeling

*N. benthamiana* seedlings constitutively expressing GFP-HDEL were stained with the following dyes and imaged with the 488 nm argon ion laser line, with emission at 500–530 nm, and the laser excitation described for each probe. For periplasmic region labeling, 2.5 mg/mL lucifer yellow CH (dipotassium salt, Sigma-Aldrich, St. Louis, MO, USA) was included in the plasmolyticum, following treatment for 45 min in 0.75 M sorbitol solution in 10 mM MES buffer without lucifer yellow to first achieve stable plasmolysis. When using the 488 nm argon ion laser line for excitation, lucifer yellow fluorescence was weaker than the relatively bright ER network, thereby providing a convenient difference in contrast that allowed distinction between the ER and the periplasm. To label the plasma membrane, 20 μM FM4-64 (ThermoFisher Scientific, Waltham MA USA) was included in the plasmolyticum during plasmolysis experiments and excited with a 543nm HeNe solid state laser, with emission at 650–750nm. For cell wall labeling, seedlings were treated with 10 μg/mL propidium iodide (PI, ThermoFisher Scientific, Waltham MA USA) for 15 min before transfer into plasmolyticum and imaged with the 543nm NeNe laser, with emission at 570–670nm.

### Confocal microscopy and image processing

Fluorescent live images were acquired using an Olympus FluoView 1000 with a numerical aperture (NA) 1.2 UPLSAPO water immersion 60x objective and an Andor laser spinning-disc confocal microscope on an inverted Olympus IX81 stand with either a 40x (NA 1.3) or 100x (NA 1.53) oil immersion objective. For persistency mapping the Olympus FluoView was used. A 70-frame time-lapse movie with frame interval 0.32 s was acquired for each region-of-interest and the first 50 frames of used for persistency mapping. Seedlings were kept stable during the period of time-lapse image recording and an auto-correlation image stabilization program was used to compensate for drift (Lucas-Kanade algorithm implemented in ImageJ, Li K. 2008. The image stabilizer plugin for ImageJ. http://www.cs.cmu.edu/~kangli/code/Image_Stabilizer.html, last accessed 4 July 2017). Persistency mapping was carried out using an in-house macro in ImageJ using the procedure described previously (Sparkes *et al.*, 2009).

The relative movement of the ER is calculated by dividing the integrated density of the sum of the displaced frame difference (DFD) by the total membrane area. The sum of the DFD is calculated by summing the difference images between every fifth frame. Total membrane area is the area of the pixels above background in the sum of all frames in the image sequence.

Organelle streaming analysis was carried out on the DIC images of the image sequence following fluorescence recovery after photobleaching (FRAP). After generating a substack of the DIC image sequence, the two fastest moving, highly refractile lipidbodies that did not move out of the frame during the analysis sequence were chosen and tracked manually by using the Manual Tracking plugin in ImageJ (Cordelières F. 2005. Manual Tracking Plugin for ImageJ, https://imagej.nih.gov/ij/plugins/track/track.html, last accessed 4 July 2017). The velocity based on the displacement between two sequential frames was calculated by the plugin, then averaged in Microsoft Excel over the entire sequence and statistics calculated.

Statistical analysis was done using the Student’s *t*-test in Microsoft Excel. Measurements for each treatment were pooled, averages were calculated and the Student’s *t*-test (*P*<0.05) used to compare each treatment by pair.

### FRAP

Photobleaching was done using the 405 nm diode SIM laser on the Olympus FluoView 100, set to 100% transmission and 10 microsecond dwell time. FRAP of ER-GFP was carried out during a 40-frame (41.4x41.4 μm, 200x200 pixel) video with a frame interval of 0.32 s. The bleach started after recording the first 10 frames using a bleach ROI of 4.14 x 4.14 μm (20 x 20 pixels). FRAP of microtubules during plasmolysis was carried out during a 20-frame (53 x 53 μm, 200 x 200 pixels) video with a frame interval of 0.8 s. The bleach started after recording the first 5 frames in a circular ROI (diameter 20 pixels). The videos were acquired as 16-bit gray-scale images. Each analyzed video was first processed with the Image stabilizer plugin of ImageJ (Li K. 2008. The image stabilizer plugin for ImageJ. http://www.cs.cmu.edu/~kangli/code/Image_Stabilizer.html). FRAP analysis was then carried out using FRAP profiler final plugin in Image J (Hardin J. 2016. FRAP profiler plugin in ImageJ. http://worms.zoology.wisc.edu/research/4d/4d.html#frap, last accessed 4 July 2017). Recovery halftime and percent mobility was calculated from the fitted curves from each sequence. For ER luminal protein photobleaching analysis, each ER component at different stages was compared by pairs using Tukey-Kramer HSD analysis, with a confidence level of 95% (*P*<0.05). For microtubule photobleaching analysis, both recovery halftime and percent mobility from each microtubule component, namely polymerized microtubules in normal cytoplasm, depolymerized microtubules in protoplast, and microtubules in Hechtian reticulum, were compared by pairs using Tukey-Kramer HSD analysis, with a confidence level of 95% (*P*<0.05).

### 3D reconstruction

Z-stack images for 3D reconstruction were taken on the Olympus FV1000 laser scanning confocal microscope with a 1.2 NA UPLSAPO water immersion 60x objective, with a step size of half of the Z-resolution. Pixel lateral dimensions were adjusted to fit the Nyquist criterion and pixel dwell time was adjusted to maximize fluorescence while maintaining fast scanning speed. 3D images were generated using surface rendering with the ImageJ 3D viewer plugin in ImageJ (Schmid B, Longair M, Schindelin J. 2013. ImageJ 3D Viewer, https://imagej.nih.gov/ij/plugins/3d-viewer/. http://3dviewer.neurofly.de, last accessed 4 July 2017) and the open source software package, Visualization Tool Kit (VTK, Kitware, Inc.) as described earlier (Enloe and Griffing, 2000). Images of 3D reconstructed structures were later exported as tiff files and edited in Adobe Illustrator CS6 ® (Adobe Systems Inc.) for labeling and annotation.

## Results

### ER undergoes increasing persistent cisternalization during osmotic shock

**Fig. 1.**
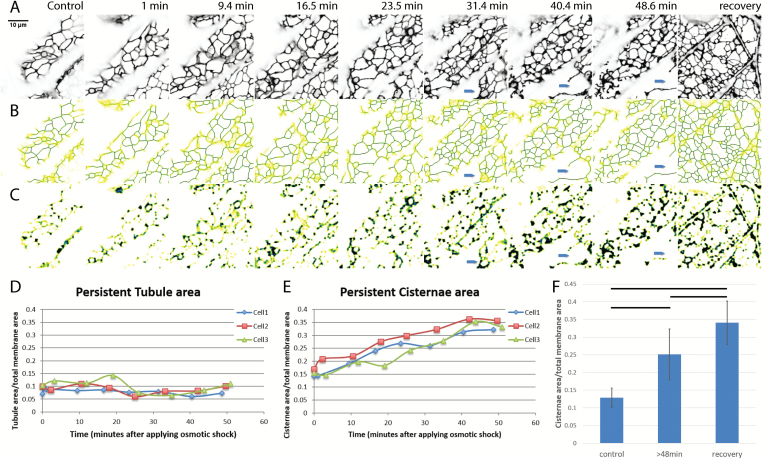
Change in amount of persistent ER cisternae and tubules after osmotic shock in *N. benthamiana* seedlings with ER lumen labeled with GFP-HDEL. A–C) Z-projections and persistency maps of time-lapse movies taken at different time points indicated by the numbers on each column showing the minutes after starting 0.75 M sorbitol treatment. Recovery is within 20 min of sorbitol wash out. Blue arrows indicate where the protoplast withdraws from the cell wall. Darker blue colors indicate the highest persistency, while yellow indicates the lowest persistency. Scale bar,10 μm. A) Projection of the sum of the movie frames showing the general structure of ER in black. B) Persistency map of ER tubules. C) Persistency map of ER cisternae. D) Tubule persistency in three different representative cells, indicated by different colors and symbols, over the times indicated after treatment with sorbitol. E) Cisternal persistency in the same three cells. F) Change in the persistent cisternal area over time as percentage of total membrane imaged. Three time points were chosen for comparison: control (before treatment), plasmolysis (after 48 min of treatment), and during recovery. Horizontal lines show significant differences with *P*<0.05. Bars show average values. Error bars are standard deviation, *n*=10 cells.

Osmotic shock carried out by treating seedlings with 0.75 M sorbitol solution in 10mM MES buffer at pH 5.8 was used to induce plasmolysis. Intact seedlings of *N. benthamiana* plants, stably expressing GFP-HDEL to label the ER, were examined before and during osmotic shock. Cortical ER of a single hypocotyl cell, specifically the fifth cell from root-shoot junction, was analyzed with persistency mapping of a time-lapse video of 70 frames. Shrinking of the entire seedling ensued within minutes of plasmolyticum addition but the shrinking rate diminished and stabilized after plasmolysis. Prior to plasmolysis, the cells have a fine polygonal, cortical ER network made up of thin tubules with a few cisternae seen at some of the tubule three-way junctions (Fig. 1A). As the protoplast withdraws from the wall during plasmolysis, usually seen by 30 min following treatment with plasmolyticum, its ER cisternalizes (Fig. 1A, C) as more persistent and non-persistent cisternae form. As early as 31 min after addition of plasmolyticum, the protoplast containing the ER pulls away from the wall (blue arrows in Fig. 1A–C). By 45 min, distinct Hechtian strands and reticula were usually seen.

At different times during osmotic shock, persistency maps of the two structural components of ER, the cisternae and tubules, were used to calculate the ratio of persistent membrane to total membrane area (Sparkes *et al.*, 2009). Both persistent tubules (Fig. 1B, D) and persistent cisternae (Fig. 1C, E) with a membrane area >0.3 μm^2^ were measured. Persistent cisterna area increased over the time course of the osmotic treatment (Fig. 1E), while the persistent tubule area remained relatively constant (Fig. 1D). Significant differences (Fig. 1F) in the increasing area of the persistent cisternae exist among cells before, during, and after the first 20 min of recovery in MES buffer. The increase in persistent cisternae is probably a combination of both a decrease in the translational movement of cisternae (see below), as well as an increase in the amount of membrane in cisternae.

**Fig. 2.**
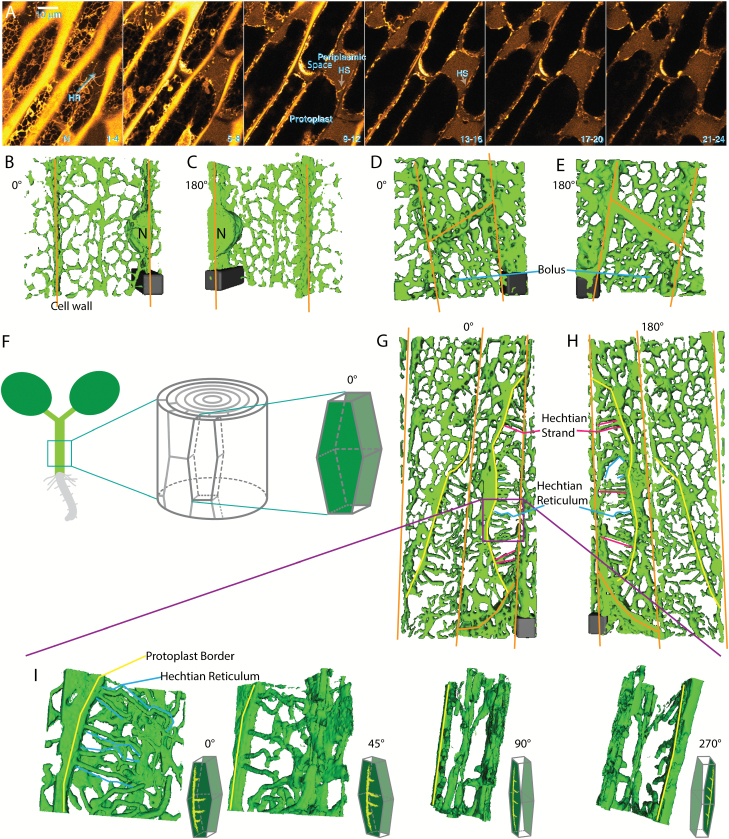
ER shape change during plasmolysis in *N. benthamiana* hypocotyl cells with ER lumen labeled with GFP-HDEL. A) Seedlings were treated for 45 min with 0.75 M sorbitol then incubated in sorbitol + 2.5 mg/mL Lucifer Yellow. Each image is the sum of four slices with a step size between slices of 0.53 μm. Slice range is indicated at bottom right of each frame. HS, Hechtian strands; HR, Hechtian reticulum; N, nucleus. Images are psuedocolored with the Orange Hot look-up table in ImageJ. Scale bar, 10 μm. The periplasmic space is indicated by the yellow regions outside the protoplasts that have a relatively lower intensity than that of the cell wall. B–C) 3D reconstruction of ER in control cells. Orange lines indicate cell wall. Scale bar, 6 μm*6 μm*15 μm. D–E) 3D reconstruction of ER during the recovery after plasmolysis. Scale bar, 6 μm*6 μm*6 μm. F) Illustration of the orientation of the hypocotyl epidermal cell used to generate 3D reconstruction. The face-on view toward the apical side wall of the epidermal cell is described as 0°. G–H) 3D reconstruction of ER during plasmolysis. Yellow lines indicate the border of withdrawing protoplasts. Representations of Hechtian strands and reticulum are indicated by magenta and blue lines, respectively. Scale bar, 6 μm*6 μm*5.7 μm. I) 3D reconstructions of ER in the specific periplasmic region in G–H (purple square) where Hechtian reticulum forms. Small inset of each frame indicates the angle of view toward the apical surface of the hypocotyl cell side wall.

The total volume of the protoplast is reduced during plasmolysis, as revealed by the filling of the periplasmic space with the diffuse fluorescence of the wall-permeant probe, Lucifer Yellow (Fig. 2A). In the periplasmic space, the remaining membranes form an altered network, the Hechtian reticulum (HR), adjacent to the wall that contains GFP-HDEL-labeled ER tubules. The wall-adjacent HR network is shown in the combined top four optical sections (first panel, sections 1–4, Fig. 2A), but is absent from the combined next four optical sections that contain the underlying periplasmic space marked with Lucifer Yellow (second panel, sections 5–8, Fig. 2A). As the cell is more deeply optically sectioned, tubules marked by the ER label occur in the periplasmic space and correspond to the Hechtian strands (HS, Fig. 2A, sections 9–12 and 13–16).

**Fig. 3.**
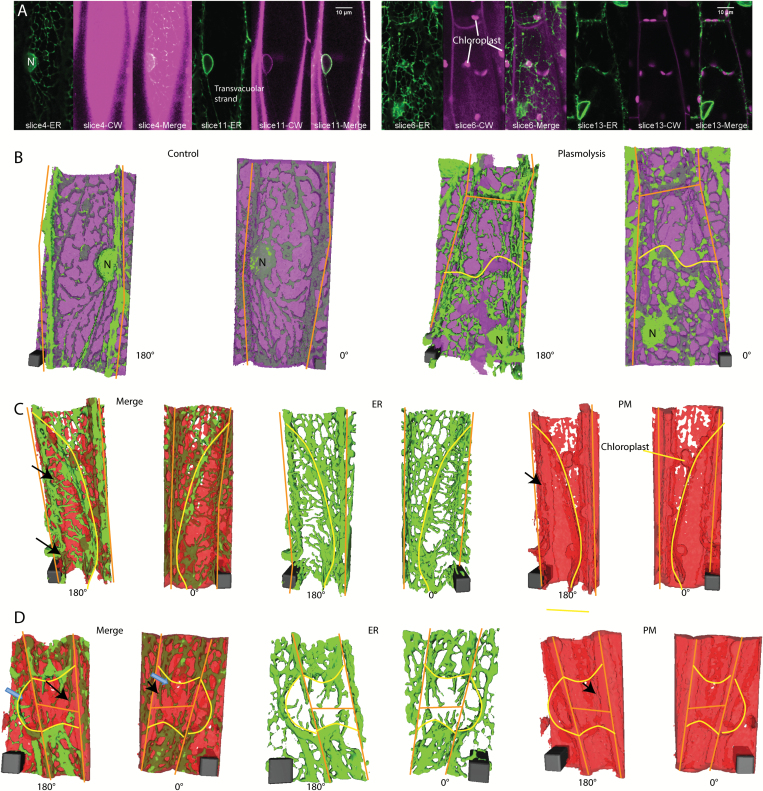
3D ER reconstructions in relation to the position of cell wall labeled with propidium iodide or plasma membrane labeled with FM4-64 in *N. benthamiana* hypocotyl cells expressing GFP-HDEL. A) Confocal images of ER (green) and cell wall labeled with propidium iodide (magenta) at two different focal planes in control (left panel) and plasmolyzed cells (right panel). B) 3D reconstructions of cells shown in (A). ER (green) and cell wall (magenta) in control (left 2 images) and plasmolyzed cells (right 2 images). Scale bar, 4μm*4μm*10.6μm for control, 4μm*4μm*14.31μm for plasmolysis. C) 3D reconstructions of a cell undergoing concave plasmolysis, showing the ER (green) and plasma membrane (red). Scale bar, 6μm*6μm*15.3μm. Black arrows indicate Hechtian strands surrounded by plasma membrane. D) 3D reconstructions of two cells undergoing convex plasmolysis and an adjacent cell undergoing concave plasmolysis, showing the ER (green) and plasma membrane (red). Black arrows indicate Hechtian reticulum with plasma membrane label on both the inside and outside. Adjacent cells often pull away in the same region but some show concave plasmolysis (blue arrow in 180° Merge) and others show convex plasmolysis (blue arrow in 0° Merge). Scale bar, 6μm*6μm*12μm. Yellow lines indicate withdrawing protoplast border, orange lines indicate cell wall border. CW, cell wall; PM, plasma membrane.

The organization of the ER-containing Hechtian reticulum and strands can be more clearly distinguished using 3D reconstructions. Figure 2B and C show reconstruction views from outside-in (0°) and from inside-out (180°), respectively, of non-plasmolyzed cells, while Fig. 2D and E show similar views of deplasmolyzed cells with increased cisternalization. Figure 2F shows how the imaged hypocotyl cells look in the context of the entire hypocotyl. Figure 2G–I show plasmolyzed cells at lower magnification (Fig. 2G, H) and higher magnification (Fig. 2I) in several different rotational angles, which display the organization of the reconstructed Hechtian reticulum and strands. In the face-on view (0°, Fig. 2I), the tubules of the Hechtian reticulum look kinked with short branches. As the view is rotated through 45° and then 90° degrees (Fig. 2I), the strands of the Hechtian reticulum foreshorten and fall in the plane just beneath the wall, while the Hechtian strands that connect the wall with the protoplast, show a continuous connection with the ER in the protoplast (Fig. 2I, 90° rotation). Rotating the view through an additional 180°, the Hechtian reticulum branches away from the observer, separate from the Hechtian strand that is connected to the protoplast ER (Fig. 2I, 270° rotation). The 3D reconstruction of the cell wall and ER confirms that the Hechtian reticulum forms at the vicinity of the inner side of cortical cell wall (Fig. 3A, B).

The formation of the Hechtian strands and Hechtian reticulum occurs in regions of both concave plasmolysis (Fig. 2G, H) and convex plasmolysis (Fig. 3A, B). The Hechtian reticulum occurs most commonly at the outer and inner periclinal walls (see Supplementary Fig. S1 at *JXB* online). Convex plasmolysis, where the protoplast forms a convex structure, often forms at the cell’s apex, while concave plasmolysis often occurs along the side of the cell. In an effort to see the plasma membrane covering the Hechtian strands, cells were labeled with the plasma membrane and endosomal dye, FM 4-64, and analyzed by 3D reconstruction. Those regions with Hechtian strands (green, continuous with ER labeling) had FM 4-64 labeled plasma membrane (red) surrounding the strands in the 180° reconstruction (black arrows in Fig. 3C, D), indicating that they are coated with plasma membrane which can be more clearly seen with electron microscopy (Oparka, 1994), whereas the Hechtian reticulum appeared to be associated with the wall-plasma membrane interface that still had FM 4–64 labeling. In regions, the Hechtian reticulum may be covered by a sheet of plasma membrane partially folding back on itself (Fig. 3C). The 3D reconstructions also show the large amount of cisternalization of the protoplast ER (Fig. 3C, D).

### Decreased movement of the ER accompanies increased cisternal persistency

**Fig. 4.**
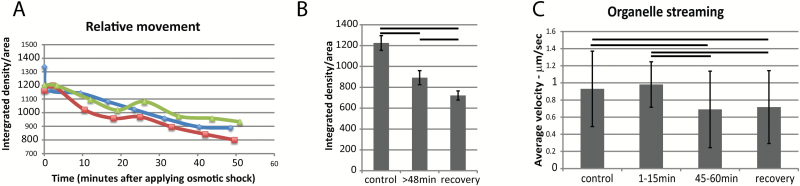
Movement of ER and other organelles during plasmolysis in *N. benthamiana* seedlings expressing GFP-HDEL. A) Movement of ER analyzed with displaced frame difference (DFD) in three separate representative cells indicated by different colors and symbols. B) Movement (DFD) of ER before and after plasmolysis and during recovery (*n*=10). Bars indicate averages and error bars represent standard deviation. Horizontal lines are between pairs that show significant differences (*P*<0.05). C) Streaming of organelles identified with DIC microscopy in the same cells analyzed for ER luminal flow (Fig. 5). Horizontal lines are between pairs that show significant differences (*P*<0.05). Bars represent averages and error bars represent standard deviation. *n*=40 for each group. (This figure is available in colour at *JXB* online)

The relative movement of the entire protoplast ER membrane is calculated from the difference in pixel intensities that occurs between every five frames, that is every 1.6 s. Those that move most show the highest integrated intensity values of the displaced frame difference, providing a pixel-based optic flow measurement of the translational movement of the ER cisternae and tubules (Griffing *et al.*, 2014). During osmotic shock, the relative movement of the total protoplast ER diminishes (Fig. 4A). There is a significant difference between the amount of relative movement of the ER before osmotic shock and 48 min after osmotic shock when it reaches stable plasmolysis (Fig. 4B). This decreased movement corresponds to increased persistent cisternalization (Fig. 1C, E). The even further reduction of ER movement in the first 20 min of recovery when plants were transferred back into pH 5.8 MES buffer without plasmolyticum (Fig. 4B) corresponds with the lack of recovery from persistent cisternalization (Fig. 2D, E).

As the ER movement diminishes during plasmolysis, so the streaming of other organelles slows. Using highly refractile 1–2 µm diameter lipid bodies identified by differential interference contrast (DIC) microscopy, the rates of streaming were analyzed before, during, and after plasmolysis, Fig. 4C. A typical streaming rate of about 1 µm/s before and during the first 15 min of plasmolysis was considerably slowed with further plasmolysis (Fig. 4C). There is a possibility that this is simply the result of artificially diminishing the cell size and that streaming rates correlate with cell size (Tominaga *et al.*, 2013; Stefano *et al.*, 2014). However, upon 20 min of recovery during deplasmolysis, when the cell resumes its previous size, the streaming rates remain low (Fig. 4C). This corresponds to the decreased movement rates of the ER (Fig. 4B).

### Changes in movement within the ER lumen during plasmolysis and deplasmolysis

Although measurement of the movement within the ER is sometimes combined with measurement of the translational movement of the ER when using some types of optic flow analysis (Ueda *et al.*, 2010; Stefano *et al.*, 2014), here we independently assess the movement within the ER lumen using FRAP. In previous studies where both FRAP and photoactivation were used to analyze the flow within the ER (Sparkes *et al.*, 2009), changes in membrane surface flow did not correlate with increased persistency of the ER. Here we examine the luminal flow of GFP-HDEL. Although ER-Golgi recycling of this luminal protein may have an effect on this flow analysis, all of the half-times of recovery are one to two orders of magnitude, namely 0.5–5 s, faster than the half-time of recovery of the ERD2-GFP marker (105 +/- 21.5 s, daSilva *et al.*, 2004). Consequently, the influence of recycling on flow rates is minimal.

**Fig. 5.**
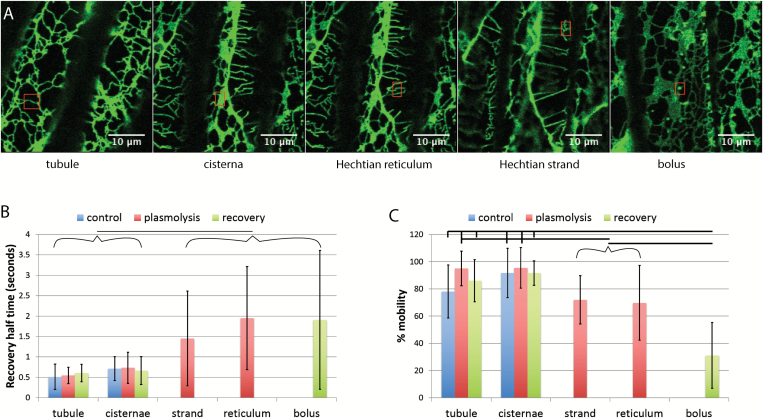
FRAP of different regions of the ER before and during plasmolysis and following recovery from plasmolysis in tobacco seedlings. A) Images showing representative regions being assessed by FRAP (red rectangles) Scale bar=10 μm. B) Recovery half-time of GFP-HDEL in tubules, cisternae, Hechtian strands and reticulum, and boluses that form upon deplasmolysis. Horizontal line between bracketed groups indicate a significant difference (*P*<0.05). *n*=18, 12, 13, 23, 9, 11, 24, 18, and 12 for each bar from left to right. C) Percentage mobility of the GFP-HDEL in tubules, cisternae, Hechtian strands and reticulum, and boluses that form upon deplasmolysis. Horizontal lines show significant differences between the indicated selected or bracketed groups (*P*<0.05, except for Hechtian strands and plasmolysis cisternae where *P*=0.0507). *n*=18, 12, 13, 23, 9, 11, 24, 18, and 13 for each bar from left to right. Bars are average values. Error bars represent standard deviation.

While plasmolysis increases cisternal persistency (Fig. 1) and decreases ER translational movement in the protoplast (Fig. 4), it does not change the half-time of fluorescence recovery or the percentage mobility of ER luminal protein in the protoplast (Fig. 5). Figure 5A and B show representative regions. Boluses of condensed GFP-HDEL form upon deplasmolysis (Fig. 5A). Experimental data from many cells indicate that the half-time for recovery does not change in the protoplast during plasmolysis but increases several fold in boluses of GFP-HDEL that form upon deplasmolysis (Fig. 5B). Likewise, the percentage mobility of GFP-HDEL does not change in the protoplast ER but there are significant decreases in the Hechtian strands, Hechtian reticulum, and boluses (Fig. 5B).

### Hechtian strands and Hechtian reticulum contain ER and appear to colocalize with the cytoskeleton in the periplasmic region

**Fig. 6.**
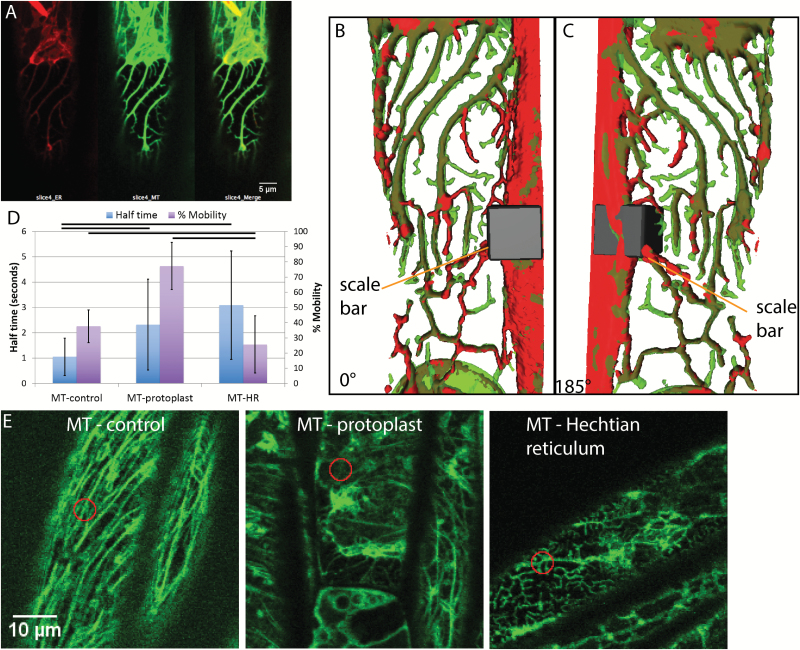
Microtubule behavior during plasmolysis in Arabidopsis hypocotyl cells with ER lumen labeled by mCherry and microtubule labeled by tubulin-GFP. A) The Hechtian reticulum of a plasmolyzed cell, showing the separate ER (red) channel, the microtubule (green) channel, and the merged image. B) 3D reconstruction of the merged volumes of the ER (red) and microtubule (green) in the Hechtian reticulum in (A) to show their colocalization. Scale bar, 7μm*7μm*10.6μm. C) 185° flip of (B). D) FRAP analysis of microtubules before plasmolysis (MT-control) and after plasmolysis in the protoplast (MT-protoplast) and in the Hechtian reticulum (MT-HR). The left-hand vertical axis shows recovery half-time; *n*=38, 49, and 55, respectively. The right-hand vertical axis shows the percentage mobility; *n*=38, 49, and 57, respectively. Horizontal bars indicate which pairwise differences are significant (*P*<0.05). Bars are averages. Error bars are standard deviation. E) Representative confocal optical sections of microtubules for FRAP analysis shown in (D). Orange circles indicate photobleached area. Scale bar, 10 μm.

The presence of microtubules in the Hechtian reticulum and strands has been previously described (Lang-Pauluzzi and Gunning, 2000; Lang *et al.*, 2014) but here we show that the ER tracks along microtubules in the Hechtian reticulum, as seen in plasmolyzed Arabidopsis hypocotyl cells that are dual-labeled with mCherry-HDEL and GFP-TUA6 (Fig. 6A–C). In the region of the Hechtian reticulum, which is formed predominately on the outer and inner periclinal walls (Supplementary Fig. S1), not only do the ER and the microtubules colocalize but the microtubules take on a branching morphology. This branching morphology differs from the normal morphology of microtubules (Fig. 6E), so an analysis of the behavior of their movements was undertaken (Fig. 6D). Although there is low percentage mobility in the control (Fig. 6D), that which is mobile shows rapid recovery, namely a half-time of 1 s, typical of the mobile unpolymerized tubulin in the background. The low mobility of the polymerized tubulin (Fig. 6D) is expected and is typical of microtubules undergoing treadmilling (Ehrhardt and Shaw, 2006). The predominance of depolymerized tubulin in the plasmolyzed protoplast shows fairly rapid and complete recovery, with a half-time of 2.3 s and a completion of 77.3% (Fig. 6D). However, FRAP dynamics of tubulin in the Hechtian reticulum differs from the other two conditions in that the tubulin has a very low percentage mobility but the part that does recover takes a longer time to do so, with a half-time of 3.1 s (Fig. 6D). This indicates that the recovery that does occur is a consequence of the movement of tubulin with a lower rate of diffusion than that found in the ‘background’ of the control.

**Fig. 7.**
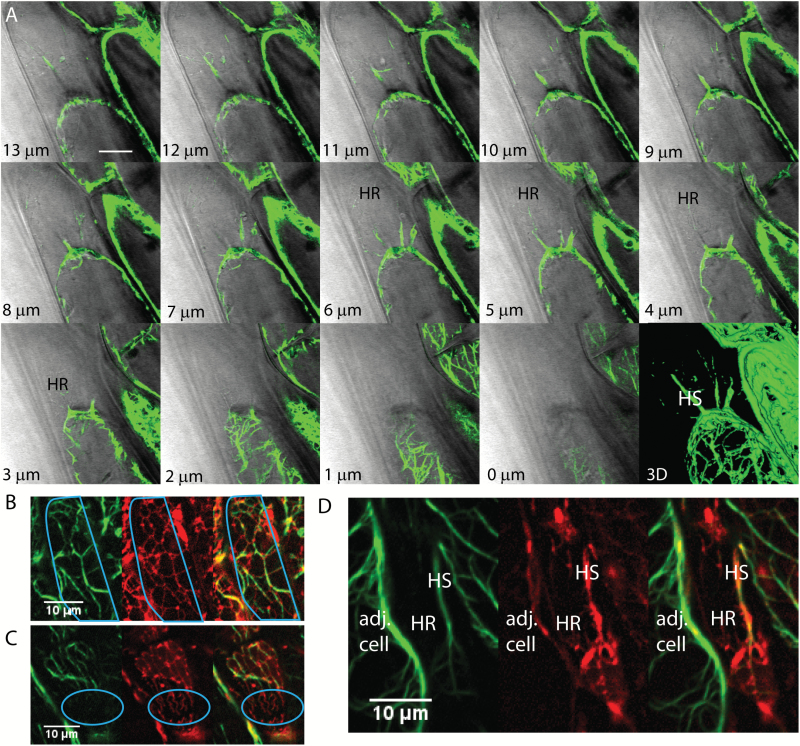
ER and actin filaments interaction during plasmolysis in Arabidopsis. A) Actin formation at different focal planes (lower left) of a plasmolyzed hypocotyl cell with a 3D reconstruction showing several Hechtian strands, one of which is labeled HS. Regions containing the Hechtian reticulum as identified with DIC are shown as HR. B–D) Confocal images of hypocotyl cells showing the separate green channel with actin filaments labeled with YFP-ABD2, the red channel showing the ER labeled with mCherry-HDEL, and a merged image. B) ER tracks on actin within the protoplast during plasmolysis. The outline of the protoplast is in cyan. C) Actin doesn’t show up in the Hechtian reticulum outlined in cyan. D) Actin forms a big Hechtian strand within the periplasmic region, labeled HS, but is excluded in the fine network of the Hechtian reticulum, labelled HR. Adj. cell, adjacent cell. Scale bar, 10 μm.

As with the microtubules, actin filaments (GFP-FABD2) were present in the Hechtian strands (Fig. 7A, D) and the polymerized actin forms a cap on the withdrawing protoplast. However, the appearance of actin in the Hechtian reticulum is more problematic. In Fig. 7B, the protoplast (outlined in cyan) ER tubules track extensively on actin. However, in the region of the Hechtian reticulum (Fig. 7C, D; outlined in cyan in Fig. 7C), the ER that is in the Hechtian reticulum is in a region devoid of actin. This is in marked contrast with the presence of tubulin in the Hechtian reticulum (Fig. 6).

## Discussion

Although the presence of the ER in components of the cell ‘left behind’ at the cell wall by the process of plasmolysis has been indicated by electron microscopy (Oparka, 1994) and vital staining (Lang-Pauluzzi and Gunning, 2000), the use of an ER-resident protein, GFP-HDEL (Sparkes *et al.*, 2009) definitively demonstrates this (Fig. 2). The ER remains attached to the PM-cell wall interface during plasmolysis and is an integral component of the Hechtian reticulum and Hechtian strands, thereby providing evidence for its strong attachment. The strength of the attachment at membrane contact sites (MCS) between the ER and the PM in plants is supported by experiments showing that ER/PM attachments persist over time (Sparkes *et al.*, 2009) and following centrifugation (Quader *et al.*, 1987).

The movement of luminal protein (GFP-HDEL) within ER is one to two orders of magnitude faster than the recycling of the putative HDEL-receptor, ERD2-GFP, from the ER through the Golgi (daSilva *et al.*, 2004). This is similar to the diffusion rate difference between YFP-KDEL and KDEL-receptor-YFP in animal cells (Nagaya *et al.*, 2008), so the flows we see are probably confined to luminal flows through the network that are relatively uninfluenced by recycling dynamics through the Golgi. In animal cells, hyperosmotic media at 960 mOsmolar does not reduce the mobile fraction of non-glycosylated YFP-KDEL, but when two glycosylation sites are engineered into YFP-KDEL, the mobile fraction is greatly diminished from 98% mobility to 38% mobility (Nagaya *et al.*, 2008). Examination of the mobility of glycosylated proteins that have an ER retention signal in plants would be of interest.

The relative luminal flow as determined by the half-time of FRAP recovery does not change in the withdrawing protoplast before, during, or after plasmolysis (Fig. 5). This is in contrast with the flow within the tubules in the Hechtian strands and reticula which change both in half-time of recovery and percentage mobility. During plasmolysis, the percentage mobility drops to about 70% in both the Hechtian strands and reticula, significantly below the near 100% in protoplast tubules and cisternae. Not only is there a smaller fraction of motile GFP-HDEL but the diffusion within the tubules slows, as shown by the doubling of the half-time for recovery in strands and tripling of the half-time for recovery in reticula. This decrease in diffusion is not surprising in strands, since they may be ‘pulled out’ and thinned by the process of plasmolysis. It has been shown that overexpression of reticulons decreases the mobility of co-expressed GFP-HDEL (Tolley *et al.*, 2008), while also producing condensed GFP-HDEL at three-way junctions and in other regions of the network. This has been interpreted to be the result of thinning the ER tubule as well. Although the strands have decreased mobility of GFP-HDEL within their lumen, the only condensed GFP-HDEL, in boluses, that we see is following deplasmolysis (Fig. 5). We interpret these boluses to be protein in isolated Hechtian strands, pulled away from the rest of the network, and upon deplasmolysis the strand either forms a vesicle or condensed GFP-HDEL and remains with limited diffusion, namely long half-times of recovery, and limited connectivity with the rest of the network, namely low percentage mobility.

**Fig. 8.**
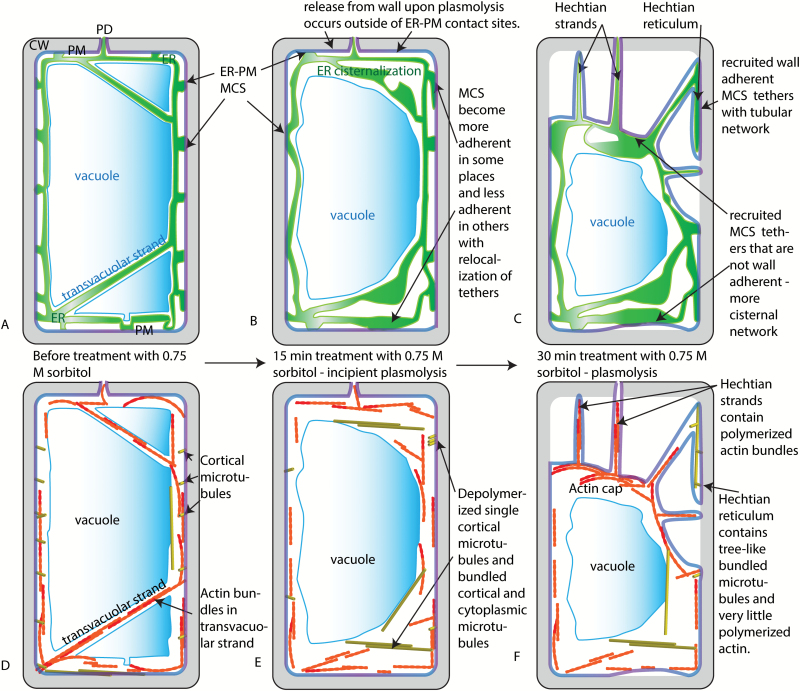
Summary and models for the change in ER and cytoskeleton during plasmolysis. A) ER, vacuole, and PM prior to plasmolysis. B) ER, vacuole, and PM after 15 min of treatment with 0.75 M sorbitol - a temporary state of incipient plasmolysis. During this time, it is proposed that an increase in cytosolic calcium is accompanied by a change in the number and distribution of MCS between the ER and the PM. It is also proposed that there is a release of the PM from the cell wall in regions enriched for MCS that are not adherent to the wall, while those ER/PM contact sites with high affinity to the wall start the process of Hechtian strand and reticulum formation. C) ER, PM, and vacuole membranes after 30–45 min in 0.75 M sorbitol. The Hechtian strands and Hechtian reticulum have formed at sites where the ER and PM and cell wall remain tethered. ER-PM contact sites on the PM of the withdrawing protoplast cause higher levels of internal cisternalization of the ER. D) Organization of microtubules and actin prior to treatment with sorbitol. E) It is proposed that an increase in cytosolic calcium ions that accompanies osmotic shock produces fewer polymerized microtubules but those that do polymerize become bundled and assume a different conformation. F) During the withdrawal of the protoplast from the cell wall after 30–45 min in 0.75 M sorbitol, actin becomes primarily associated with Hechtian strands and not the Hechtian reticulum. An actin cap forms on the withdrawing protoplast. Meanwhile, the microtubules reform as macrotubulin-containing tree-like structures in the Hechtian reticulum. ER, endoplasmic reticulum; CW, cell wall; MCS, membrane contact sites; PD, plasmodesma; PM, plasma membrane.

The situation for luminal flow in the Hechtian reticulum is quite different. Since it is not stretched like the ER in the Hechtian strand, there is no *a priori* reason to think that it is thinned. However, its half-time of recovery is even longer than that in the Hechtian strands; hence, apparent diffusion is considerably reduced. This may be a consequence of being encased in the folds of the plasma membrane, which may wrap around it. In that situation, the ER might have at least two sides of its membrane bound to the plasma membrane (Fig. 8C) and that may alter the flow within the lumen if surface flow is linked to luminal flow.

Several proteins that have been shown to be present at or near the ER/PM MCS, namely Vamp-associated protein 27 (VAP27), NET3C (Wang *et al.*, 2014) and synaptotagmin 1 (Syt 1; Pérez-Sancho *et al.*, 2015; Levy *et al.*, 2015) may act as tethers between the ER and the PM. Syt 1 is an ER-resident protein that is similar to extended synaptotagmins (E-Syts). E-Syts have extended calcium-sensitive domains that bind the ER to the PM (Giordano *et al.*, 2013). When calcium re-uptake by the ER is inhibited in animal cells (Poteser *et al.*, 2016), with the concomitant increase in cytosolic calcium (Giordano *et al.*, 2013), ER/PM MCS increase (Poteser *et al.*, 2016) and ER-localized Syt1 is recruited to ER/PM MCS (Giordano *et al.*, 2013). As cytosolic calcium increases upon hyperosmotic treatment (Choi *et al.*, 2014), it can be hypothesized that this changes the nature of the PM/ER MCS in plants (Fig. 8B, C) after it reaches a certain critical level, as it does in animal cells. The generation of more extensive PM/ER MCS associated with regions of the PM that have low affinity for the cell wall is consistent with the presence of persistent, that is probably associated with the PM, cisternal ER structures seen in the withdrawing protoplast (Figs 1 and 8C).

A likely function of MCS may be to generate subdomains that compartmentalize the biochemical activity of the ER and PM (Dittman and Menon, 2017). Furthermore, there appear to be MCS ‘master switches’ that can change the abundance of some MCS subdomains relative to other MCS subdomains (Elbaz-Alon *et al*., 2015). Two separate tethers, Syt1 and VAP27, have recently been shown to be in separate MCS subdomains in Arabidopsis (Siao *et al*., 2016). The presence of ER in Hechtian strands and reticula would be consistent with a separate subdomain of the ER/PM that has high affinity for the cell wall (Fig. 8C). With increasing calcium, a separate set of ER/PM tether(s) would be recruited to a subdomain of the PM with high affinity to the wall and the tubules would thereby be stabilized at the cell cortex. The nature of the connection between the cell wall, the PM, and the ER is not known, but we suggest that it becomes more adherent in these regions of tubular ER, which then form the Hechtian reticula and Hechtian strands. As described below and in Fig. 8E and F, the cortical microtubules are also stabilized in this region and undergo some transformation (Fig. 6).

During plasmolysis, cortical microtubules depolymerize and bundle in the withdrawing protoplast (Fig. 6E). However tubulin, in a different state as determined by FRAP, is left behind in the Hechtian reticulum (Fig. 6D, E). These are not the treadmilling polymers in the cortex of non-plasmolyzed cells. No individual microtubules are seen treadmilling; instead, relatively non-mobile bundles or ‘trees’ of tubulin form, some of which is slowly diffusively exchangeable, with a large half-time of recovery from FRAP (Fig. 6D). These tubulin ‘trees’ have been seen before using immunocytochemistry of the fixed Hechtian reticulum (Lang-Pauluzzi, 2000; Lang-Pauluzzi and Gunning, 2000) or GFP-tubulin cell lines (Lang *et al.*, 2014). Interestingly, as shown here, the ER in the Hechtian reticulum tracks the tubulin ‘trees’. The general depolymerization of microtubules in the retracting protoplast, which we speculate to be driven by increased cytoplasmic calcium ion concentration, is accompanied by the appearance of these wavy, treelike, bundled polymers (Fig. 8E, F).

The nature of this tubulin is unclear but we can rule out the possibility that it is all completely depolymerized and trapped in pockets of folded over plasma membrane encasing it as there is little mobility of the tubulin, as determined by FRAP (Fig. 6). It could be depolymerized but have low mobility because it is bound tightly to the plasma membrane. Alternatively, it could be polymerized in a different state than normal microtubules. In this case, the tubulin would be bound to other tubulin subunits but in a way that allows local diffusion and substitution of monomers, unlike normal microtubules. Such microtubules have previously been detected after plasmolysis and are called ‘macrotubules’, which apparently assemble differently from normal microtubules because they have a larger diameter in electron micrographs (Komis *et al.*, 2002*a*).

The extensive and dynamic tubular reticulum in the retracting protoplast is correlated with an active actin network (Fig. 7). Also indicative of an active actin network is the maintenance of cytoplasmic streaming in the plasmolyzing protoplast (Fig. 4). The association of actin with the PM on the retracting protoplast is strong in areas that are separated from the cell wall, forming a cap of actin on apically plasmolyzing cells (Fig. 7). Actin filament bundles are common in large Hechtian strands and in cytoplasmic connections between subprotoplasts (Komis *et al*., 2002*b*, 2003; Lang *et al.*, 2014), perhaps serving a stabilizing role. These regions, however, are also not in direct contact with the cell wall. The absence of detectable actin in the Hechtian reticulum when actin-associated proteins are present in some MCS (NET3C, Wang *et al*., 2014) would be consistent with the model that different MCS subdomains separate upon plasmolysis, as described above. The actin-containing MCS would be those giving rise to persistent cisternae in the protoplast, while the tubulin-associated MCS would be those in the Hechtian reticulum. Unlike the cisternalization that occurs in the presence of latrunculin or myosin dominant negative experiments (Sparkes *et al.*, 2009), it is not accompanied by increased persistent tubulation (Fig. 1A), thereby supporting the conclusion that the persistent cisternalization caused by plasmolysis is different from that caused by inactivation of the actin-based cytoskeleton.

In summary, plasmolysis reveals different kinds of association between the ER with the PM. The PM of the withdrawing protoplast is associated with persistent ER cisternae and actin. The PM of the Hechtian reticulum is associated with the cell wall and with persistent ER tubules that track along macrotubular tubulin. The mobility of luminal GFP-HDEL is different in these two ER/PM associations. Our model is that these MCS subdomains separate when hyperosmotic treatment causes a calcium influx. The separation of the subdomains may be achieved through the differential calcium binding of ER tethers to the PM. This model can be tested in the future by examining cytoplasmic calcium during plasmolysis as the protoplast withdraws from the cell wall and by analyzing the subcellular distribution of ER/PM MCS characterized by separate tether populations following plasmolysis.

## Supplementary data

Supplementary data are available at *JXB* online.

Fig. S1. Hechtian reticulum forms extensively against outer and inner periclinal walls.

## Supplementary Material

Supplementary Figure_S1

## Abbreviations:

ER: endoplasmic reticulum
FRAP: fluorescence recovery after photobleaching
HR: Hechtian reticulum
HS: Hechtian strands
MCS: membrane contact sites
MES: 2-(N-morpholino) ethanesulfonic acid
PM: plasma membrane.

## References

[CIT0001] Choi WG, Toyota M, Kim SH, Hilleary R, Gilroy S 2014 Salt stress-induced Ca2+ waves associated with rapid, long-distance root-to-shoot signaling in plants. Proceedings of the National Academy of Sciences, USA 111, 6497–6502. 10.1073/pnas.1319955111 PMC4035928 24706854

[CIT0002] daSilva LL, Snapp EL, Denecke J, Lippincott-Schwartz J, Hawes C, Brandizzi F 2004 Endoplasmic reticulum export sites and Golgi bodies behave as single mobile secretory units in plant cells. The Plant Cell 16, 1753–1771. 15208385 10.1105/tpc.022673 PMC514159

[CIT0003] Dittman JS, Menon AK 2017 Speed limits for nonvesicular intracellular sterol transport. Trends in Biochemical Sciences 42, 90–97. 27956059 10.1016/j.tibs.2016.11.004 PMC5272819

[CIT0004] Ehrhardt DW, Shaw SL 2006 Microtubule dynamics and organization in the plant cortical array. Annual Review of Plant Biology 57, 859–875. 10.1146/annurev.arplant.57.032905.105329 16669785

[CIT0005] Elbaz-Alon Y, Eisenberg-Bord M, Shinder V, Stiller SB, Shimoni E, Wiedemann N, Geiger T, Schuldiner M 2015 Lam6 regulates the extent of contacts between organelles. Cell Reports 12, 7–14. 26119743 10.1016/j.celrep.2015.06.022 PMC4518459

[CIT0006] Enloe L, Griffing L 2000 Improved volume rendering for the visualization of living cells examined with confocal microscopy. Procedings of the SPIE 3960, 385–392.

[CIT0007] Giordano F, Saheki Y, Idevall-Hagren O 2013 PI(4,5)P(2)-dependent and Ca(2+)-regulated ER-PM interactions mediated by the extended synaptotagmins. Cell 153, 1494–1509. 23791178 10.1016/j.cell.2013.05.026 PMC3716012

[CIT0008] Griffing LR, Gao HT, Sparkes I 2014 ER network dynamics are differentially controlled by myosins XI-K, XI-C, XI-E, XI-I, XI-1, and XI-2. Frontiers in Plant Science 5, 218. 24904614 10.3389/fpls.2014.00218 PMC4033215

[CIT0009] Griffing LR, Lin C, Perico C, White RR, Sparkes I 2016 Plant ER geometry and dynamics: biophysical and cytoskeletal control during growth and biotic response. Protoplasma 254, 43–56. 26862751 10.1007/s00709-016-0945-3 PMC5216105

[CIT0010] Hamada T, Ueda H, Kawase T, Hara-Nishimura I 2014 Microtubules contribute to tubule elongation and anchoring of endoplasmic reticulum, resulting in high network complexity in Arabidopsis. Plant Physiology 166, 1869–1876. 25367857 10.1104/pp.114.252320 PMC4256883

[CIT0011] Haseloff J, Simerling KR, Prasher DC, Hodge S 1997 Removal of a cryptic intron and subcellular localization of green fluorescent protein are required to mark transgenic Arabidopsis plants brightly. Proceedings of the National Academy of Sciences, USA 94, 2122–2127. 10.1073/pnas.94.6.2122 PMC20051 9122158

[CIT0012] Hecht K 1912 Studien über den Vorgang der Plasmolyse. Beiträge zur Biologie der Pflanzen 11, 133–189.

[CIT0013] Hu J, Prinz WA, Rapoport TA 2012 Weaving the web of ER tubules. Cell 148, 832–832. 10.1016/j.cell.2011.11.022 PMC3478066 22153070

[CIT0014] Komis G, Apostolakos P, Galatis B 2002a Hyperosmotic stress induces formation of tubulin macrotubules in root-tip cells of *Triticum turgidum*: Their probable involvement in protoplast volume control. Plant and Cell Physiology 43, 911–922. 12198194 10.1093/pcp/pcf114

[CIT0015] Komis G, Apostolakos P, Galatis B 2002b Hyperosmotic stress-induced actin filament reorganization in leaf cells of *Chlorophyton comosum*. Journal of Experimental Botany 53, 1699–1710. 12147720 10.1093/jxb/erf018

[CIT0016] Komis G, Apostolakos P, Galatis B 2003 Actomyosin is involved in the plasmolytic cycle: gliding movement of the deplasmolyzing protoplast. Protoplasma 221, 245–256. 12802632 10.1007/s00709-002-0054-3

[CIT0017] Lang-Pauluzzi I 2000 The behaviour of the plasma membrane during plasmolysis: a study by UV microscopy. Journal of Microscopy 198, 188–198. 10849197 10.1046/j.1365-2818.2000.00677.x

[CIT0018] Lang-Pauluzzi I, Gunning BES 2000 A plasmolytic cycle: the fate of cytoskeletal elements. Protoplasma 212, 174–185.

[CIT0019] Lang I, Sassmann S, Schmidt B, Komis G 2014 Plasmolysis: Loss of turgor and beyond. Plants 3, 583–593. 27135521 10.3390/plants3040583 PMC4844282

[CIT0020] Levy A, Zheng JY, Lazarowitz SG 2015 Synaptotagmin SYTA forms ER-plasma membrane junctions that are recruited to plasmodesmata for plant virus movement. Current Biology 25, 2018–2025. 26166780 10.1016/j.cub.2015.06.015 PMC4526382

[CIT0021] Nagaya H, Tamura T, Higa-Nishiyama A 2008 Regulated motion of glycoproteins revealed by direct visualization of a single cargo in the endoplasmic reticulum. The Journal of Cell Biology 180, 129–143. 18195104 10.1083/jcb.200704078 PMC2213621

[CIT0022] Nobel PS 2009 Physicochemical and environmental plant physiology, Ed 4 UK: Elsevier Inc, Linacre House, Jordan Hill, Oxford OX2 8DP

[CIT0023] Oparka K 1994 Plasmolysis: New insights into an old process. New Phytologist 126, 571–591

[CIT0024] Oparka KJ, Prior DAM, Crawford JW 1994 Behaviour of plasma membrane, cortical ER and plasmodesmata during plasmolysis of onion epidermal cells. Plant, Cell and Environment 17, 163–171.

[CIT0025] Park E, Nebenführ A 2013 Myosin XIK of *Arabidopsis thaliana* accumulates at the root hair tip and is required for fast root hair growth. PLoS One 8, e76745. 24116145 10.1371/journal.pone.0076745 PMC3792037

[CIT0026] Perez-Sancho J, Vanneste S, Lee E, McFarlane HE, Esteban Del Valle A, Valpuesta V, Friml J, Botella MA, Rosado A 2015 The Arabidopsis synaptotagmin1 is enriched in endoplasmic reticulum-plasma membrane contact sites and confers cellular resistance to mechanical stresses. Plant Physiology 168, 132–143. 25792253 10.1104/pp.15.00260 PMC4424031

[CIT0027] Poteser M, Leitinger G, Pritz E, Platzer D, Frischauf I, Romanin C, Groschner K 2016 Live-cell imaging of ER-PM contact architecture by a novel TIRFM approach reveals extension of junctions in response to store-operated Ca2+-entry. Science Reports 6, 35656. 10.1038/srep35656 PMC5069484 27759093

[CIT0028] Quader H, Hofmann A, Schnepf E 1987 Shape and movement ofthe endoplasmic reticulum in onion bulb epidermis cells: possible involvement of actin. European Journal of Cell Biology 44, 17–26.

[CIT0029] Ridge RW, Uozumi Y, Plazinski J, Hurley UA, Williamson RE 1999 Developmental transitions and dynamics of the cortical ER of Arabidopsis cells seen with green fluorescent protein. Plant and Cell Physiology 40, 1253–1261. 10682347 10.1093/oxfordjournals.pcp.a029513

[CIT0030] Ruiz MT, Voinnet O, Baulcombe DC 1998 Initiation and maintenance of virus-induced gene silencing. The Plant Cell 10, 937–946. 9634582 10.1105/tpc.10.6.937 PMC144041

[CIT0031] Runions J, Brach T, Kuhner S, Hawes C 2006 Photoactivation of GFP reveals protein dynamics within the endoplasmic reticulum membrane. Journal of Experimental Botany 57, 43–50. 16207749 10.1093/jxb/eri289

[CIT0032] Siao W, Wang P, Voigt B, Hussey PJ, Baluska F 2016 Arabidopsis SYT1 maintains stability of cortical endoplasmic reticulum networks and VAP27-1-enriched endoplasmic reticulum-plasma membrane contact sites. Journal of Experimental Botany 67, 6161–6171. 27811083 10.1093/jxb/erw381 PMC5100027

[CIT0033] Sparkes I, Runions J, Hawes C, Griffing L 2009 Movement and remodeling of the endoplasmic reticulum in nondividing cells of tobacco leaves. The Plant Cell 21, 3937–3949. 20040535 10.1105/tpc.109.072249 PMC2814503

[CIT0034] Stefano G, Renna L, Brandizzi F 2014 The endoplasmic reticulum exerts control over organelle streaming during cell expansion. Journal of Cell Science 127, 947–953. 24424025 10.1242/jcs.139907

[CIT0035] Terasaki M 2000 Dynamics of the endoplasmic reticulum and Golgi apparatus during early sea urchin development. Molecular Biology of the Cell 11, 897–914. 10712508 10.1091/mbc.11.3.897 PMC14819

[CIT0036] Terasaki M, Loew L, Lippincott-Schwartz J, Zaal K 2001 Fluorescent staining of subcellular organelles: ER, Golgi complex, and mitochondria. Current Protocols in Cell Biology Chapter 4, Unit 4–4. 10.1002/0471143030.cb0404s00 18228364

[CIT0037] Tolley N, Sparkes IA, Hunter PR, Craddock CP, Nuttall J, Roberts LM, Hawes C, Pedrazzini E, Frigerio L 2008 Overexpression of a plant reticulon remodels the lumen of the cortical endoplasmic reticulum but does not perturb protein transport. Traffic 9, 94–102. 17980018 10.1111/j.1600-0854.2007.00670.x

[CIT0038] Tominaga M, Kimura A, Yokota E, Haraguchi T, Shimmen T, Yamamoto K, Nakano A, Ito K 2013 Cytoplasmic streaming velocity as a plant size determinant. Developmental Cell 27, 345–352. 24229646 10.1016/j.devcel.2013.10.005

[CIT0039] Ueda H, Yokota E, Kutsuna N, Shimada T, Tamura K, Shimmen T, Hasezawa S, Dolja VV, Hara-Nishimura I 2010 Myosin-dependent endoplasmic reticulum motility and F-actin organization in plant cells. Proceedings of the National Academy of Science, USA 107, 6894–6899. 10.1073/pnas.0911482107 PMC2872430 20351265

[CIT0040] Ueda K, Matsuyama T, Hashimoto T 1999 Visualization of microtubules in living cells of transgenic *Arabidopsis thaliana*. Protoplasma 206, 201–206.

[CIT0041] Voigt B, Timmers ACJ, Samaj J, Muller J, Baluska F, Menzel D 2005 GFP-FABD2 fusion construct allows in vivo visualization of the dynamic actin cytoskeleton in all cells of Arabidopsis seedlings. European Journal of Cell Biology 84, 595–608. 16032928 10.1016/j.ejcb.2004.11.011

[CIT0042] Wang P, Hawkins TJ, Richardson C, Cummins I, Deeks MJ, Sparkes I, Hawes C, Hussey PJ 2014 The plant cytoskeleton, NET3C, and VAP27 mediate the link between the plasma membrane and endoplasmic reticulum. Current Biology 24, 1397–1405. 24909329 10.1016/j.cub.2014.05.003

