# Supplementary figures and images for: Plasmolysis-deplasmolysis causes changes in endoplasmic reticulum form, movement, flow, and cytoskeletal association
Source: J Exp Bot. 2017 Aug 23;68(15):4075–87. doi: 10.1093/jxb/erx243 (PMC5853952; doi:10.1093/jxb/erx243)

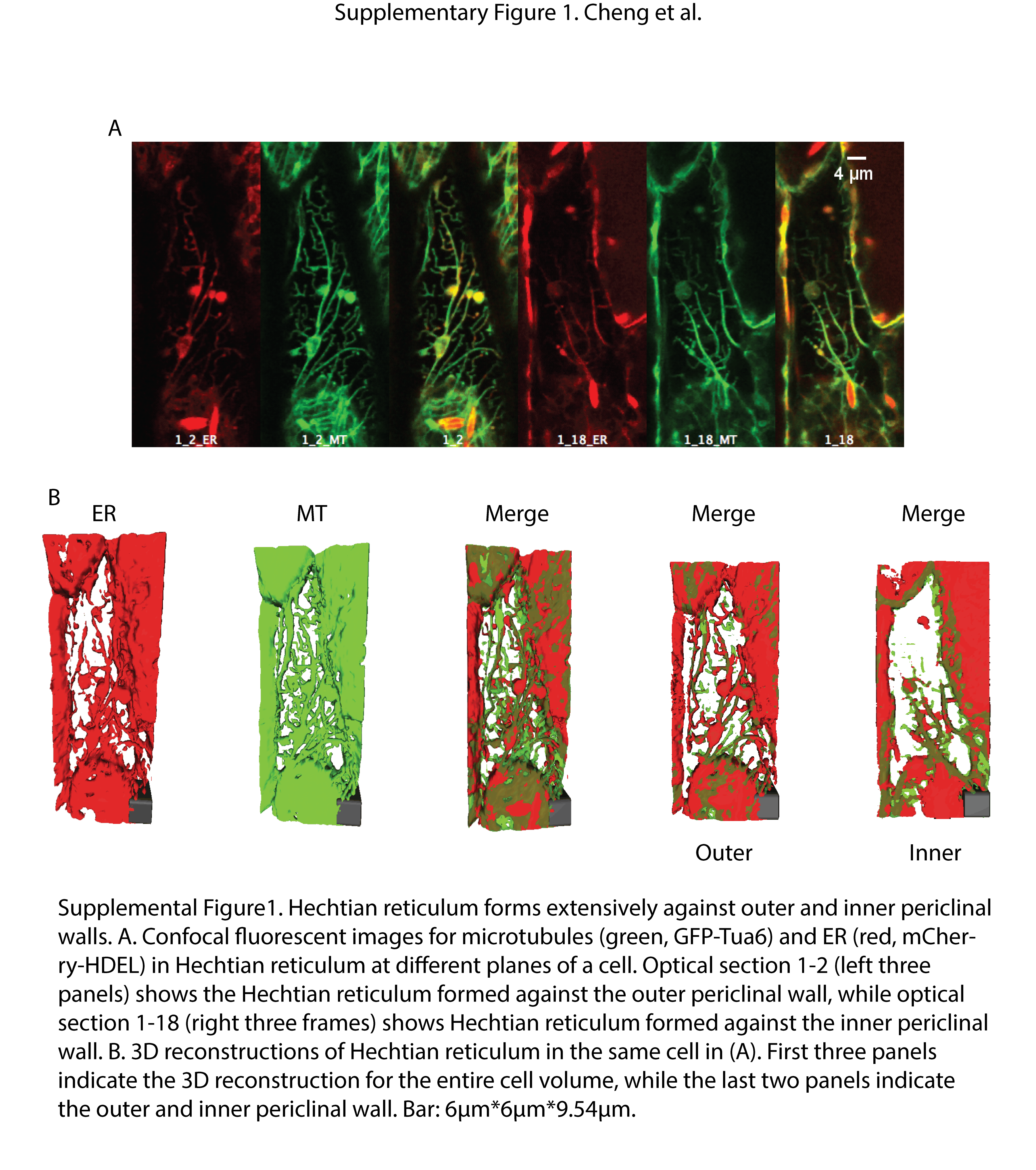

Supplement: Supplementary Figure_S1 [file erx243_suppl_supplementary_figure_s1.png]
